# Modulation of angiotensin II signaling in the prevention of fibrosis

**DOI:** 10.1186/s13069-015-0023-z

**Published:** 2015-04-23

**Authors:** Amanda M Murphy, Alison L Wong, Michael Bezuhly

**Affiliations:** Division of Plastic and Reconstructive Surgery, Dalhousie University, 5850/5980 University Avenue, PO Box 9700, B3K 6R8 Halifax, NS Canada; IWK Health Centre, Dalhousie University, 5850/5980 University Avenue, PO Box 9700, B3K 6R8 Halifax, NS Canada

## Abstract

Over the last decade, it has become clear that the role of angiotensin II extends far beyond recognized renal and cardiovascular effects. The presence of an autologous renin-angiotensin system has been demonstrated in almost all tissues of the body. It is now known that angiotensin II acts both independently and in synergy with TGF-beta to induce fibrosis via the angiotensin type 1 receptor (AT_1_) in a multitude of tissues outside of the cardiovascular and renal systems, including pulmonary fibrosis, intra-abdominal fibrosis, and systemic sclerosis. Interestingly, recent studies have described a paradoxically regenerative effect of the angiotensin system via stimulation of the angiotensin type 2 receptor (AT_2_). Activation of AT_2_ has been shown to ameliorate fibrosis in animal models of skeletal muscle, gastrointestinal, and neurologic diseases. Clinical reports suggest a beneficial role for modulation of angiotensin II signaling in cutaneous scarring. This article reviews current knowledge on the role that angiotensin II plays in tissue fibrosis, as well as current and potential therapies targeting this system.

## Review

Angiotensin II (AngII) has long been recognized as the principal vasoactive mediator of the renin-angiotensin system (RAS). Over the past 20 years, however, it has become clear that effects of AngII extend beyond systemic cardiovascular and renal actions. Once believed to act only as a circulating hormone, it is now evident that a local ‘tissue RAS’ exists in almost all organs and tissues, including the heart [1], blood vessels [2], brain [2], kidney [2], fat [3], liver [4], and skin [5]. Tissue RASs are functionally autonomous systems that have been shown to play an important role in the development of fibrosis [6,7]. The following is a review of the role of AngII in fibrotic disorders, from cellular signaling to clinical correlation, and potential therapies aimed at modulating the angiotensin pathway beyond the cardiovascular and renal systems.

### General mechanisms of fibrosis

The mammalian response to injury occurs in three distinct phases [8]. The initial inflammatory phase occurs immediately following insult and involves activation of the coagulation cascade, fibrin deposition, and infiltration of macrophages and neutrophils [9]. Following this, the proliferative phase is defined by angiogenesis, fibroblast proliferation, and differentiation [10,11]. Finally, in the remodeling phase, fibroblasts and myofibroblasts deposit a collagen-rich extracellular matrix that will ultimately become a scar that replaces the injured functional tissue. This process of healing is highly conserved across tissue types.

Fibrosis also occurs ubiquitously throughout the body as a pathologic response to chronic tissue injury and is essentially a persistence of the normal wound healing response. It is characterized by chronic inflammation and persistence of myofibroblasts ultimately resulting in excess accumulation of extracellular matrix and destruction of the normal tissue architecture. Fibrogenesis is most likely triggered by inflammation, whether or not inflammation leads to tissue repair or to fibrosis depending on the balance between extracellular matrix (ECM) synthesis and degradation [12]. In response to chronic tissue damage, ECM-producing cells, namely fibroblasts, undergo a process of activation characterized by proliferation and differentiation into myofibroblasts, which are the main cellular effectors of fibrosis [13]. These cells deposit large amounts of ECM proteins and express the contractile protein α-smooth muscle actin (α-SMA), which contributes to the decreased tissue compliance associated with fibrosis. This activation is regulated by several soluble factors including cytokines, growth factors, and products of oxidative stress [14,15]. Although several molecules are involved in this process, transforming growth factor-beta 1 (TGF-β1) plays a pivotal role in triggering and sustaining fibrogenesis [16].

### Renin-angiotensin system

In the classical description, decreased renal perfusion results in renin release from the juxtaglomerular cells of the kidney. This enzyme then cleaves angiotensinogen, produced in the liver, to angiotensin I which is subsequently converted to angiotensin II by angiotensin-converting enzyme (ACE) in the lung. AngII acts systemically to regulate blood pressure as well as water and electrolyte homeostasis [17].

We now know that AngII is expressed and acts on almost all tissues. AngII signals through two main receptors, angiotensin receptor 1 (AT_1_) and angiotensin receptor 2 (AT_2_) [7]. AT_1_ mediates the ‘classical’ effects of AngII, namely regulation of blood pressure as well as sodium and water homeostasis. Locally, AT_1_ activation stimulates profibrotic downstream effects, namely inflammatory cell recruitment, angiogenesis, cellular proliferation, and accumulation of ECM [6,7].

Until recently, the role of AT_2_ was less well defined; however, recent research has revealed that AT_2_ receptor stimulation counteracts the harmful effects of AT_1_ signaling in fibrotic disease [18-20]. The so-called ‘protective arm’ of RAS has expanded to include the heptapeptide angiotensin 1 to 7 (Ang1-7), its receptor, Mas, and angiotensin-converting enzyme 2 (ACE2), which produces Ang1-7 from both AngI and AngII [21,22]. Activation of these counterregulatory systems has been shown to decrease inflammation, cell proliferation, and collagen deposition [21,23] and improve healing in experimental models of the cardiovascular, renal, immunologic, and neurologic systems [24]. Although studies have shown that both AT_1_ and AT_2_ receptors are upregulated after injury [23], the interplay between the two remains to be elucidated, particularly with regard to AT_2_. At present, mechanisms responsible for the regenerative effects associated with AT_2_ remain unclear. There is evidence that the anti-inflammatory effects of AT_2_ are in part related to inhibition of NF-κΒ-mediated transcription of inflammatory mediators; however, the downstream signaling effects remain a focus of ongoing research [19].

### Angiotensin-TGF-β signaling

**Figure 1:**
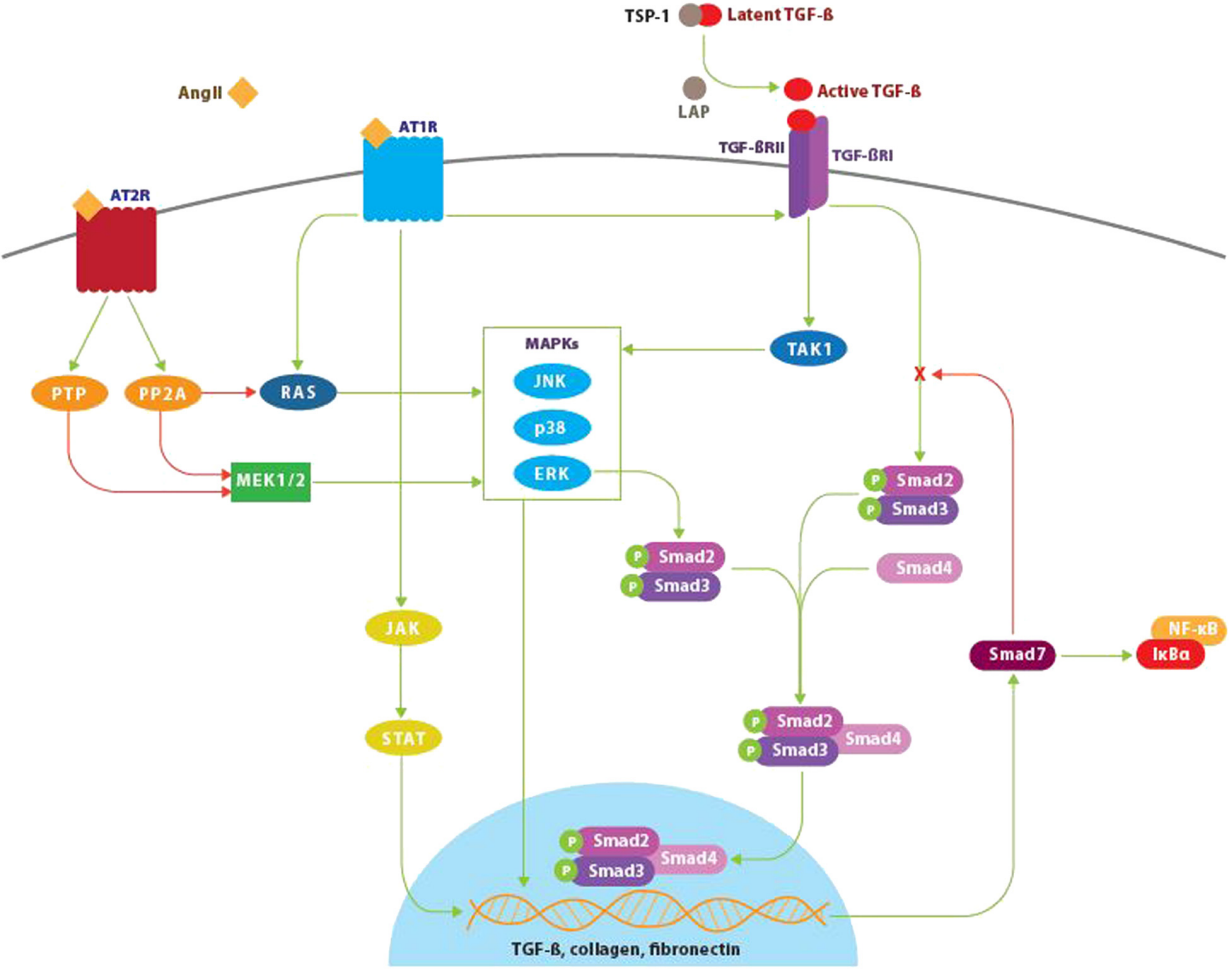
Angiotensin II, transforming growth factor-β, and Smad signaling pathways. Binding of angiotensin II (AngII) to the angiotensin type 1 receptor (AT_1_) results in Smad2 and Smad3 phosphorylation via the ERK/p38/MAPK pathway. Activated Smad2 and Smad3 complex with Smad4 and translocate into the nucleus resulting in transcription of target genes including transforming growth factor-β (TGF-β), procollagen I, procollagen III, and fibronectin. AngII-AT_1_ binding also directly activates TGF-β, which in turn activates Smad signaling in a similar manner. The P-Smad2/3-Smad4 complex induces transcription of Smad7, which has an inhibitory effect on TGF-β by targeting TGF-β receptor I (TGF-βR1) and Smads for ubiquitin-dependent degradation. Smad7 also inhibits NF-κB-driven inflammation via induction of the NF-κB inhibitor, IκBα. Conversely, activation of the AngII type 2 receptor (AT_2_) signaling has an inhibitory effect on both Smad and MAPK signaling pathways via dephosphorylating actions of phosphotyrosine phosphatase and protein phosphatase 2A. This produces antiproliferative and survival-promoting effects that oppose AT_1_-mediated fibrotic changes. Green lines indicate positive regulation. Red lines indicate negative regulation. Latent TGF-β binding protein (LTBP), TGF-β receptor 2 (TGF-βIIR), thrombospondin-1 (TSP-1), extracellular signal-relate kinase (ERK), nuclear factor kappa-light-chain-enhancer of activated B cells (NF-κB), inhibitor of kappa B alpha (IκBα), protein serine/threonine phosphatase 2A (PP2A), phosphotyrosine phosphatase (PTP).

Despite the reported anti-inflammatory effects of the AT_2_ and Mas counter-regulatory systems, current research has continued to focus on the predominantly profibrotic role of AngII. The fibrogenic effects of AngII have been linked to its activation of TGF-β1 signaling (Figure 1) [25-27]. The TGF-β family has been extensively studied and is known to be involved in a variety of cellular functions, both physiologic and pathologic [28]. They are known to critically regulate tissue homeostasis and repair, immune and inflammatory responses, ECM deposition, cell differentiation and growth [29,30]. In mammals, three isoforms exist, designated TGF-β1, TGF-β2, and TGF-β3, with TGF-β1 being the most prevalent and expressed in almost all tissues [30]. Overexpression of TGF-β1 has been established as a key contributor to fibrosis in almost all tissues [16,25]. It potently stimulates myofibroblast differentiation and synthesis of ECM proteins [31]. TGF-β1 also acts to preserve ECM proteins by inhibiting the activity of matrix metalloproteinases (MMPs) and inducing synthesis of tissue inhibitor metalloproteinases (TIMPs) [32]. Additionally, it strongly induces connective tissue growth factor (CTGF), a critical profibrotic mediator implicated in fibroblast proliferation, cellular adhesion, and ECM synthesis [33].

TGF-β1 is secreted as a latent precursor, bound to TGF-β1 latency-associated peptide (LAP), and requires proteolytic cleavage to become active [34]. Significant amounts of latent TGF-β1 are stored in the ECM of most tissues; however, activation of just a small fraction of latent TGF-β1 elicits maximal cellular response [35]. Following activation, TGF-β1 binds to the TGF-β1 type II receptor (TβRII), which subsequently complexes with and transphosphorylates the TGF-β1 type I receptor (TβRI), also known as ALK5 [36]. Active TβRI in turn activates the intracellular Smad signaling cascade by phosphorylating Smad2 and Smad3. Smad2 and Smad3 complex with Smad4 and translocate to the nucleus where they activate genes which code for extracellular matrix proteins including type I collagen and fibronectin. Activation of Smad3 also induces expression of Smad7, which inhibits TGF-β1-mediated effects, forming a negative feedback loop [30].

In addition to activation of Smad signaling, TGF-β1 can also activate several noncanonical mitogen-activated protein kinase pathways, namely extracellular signal-related kinase (ERK), p38 MAPK, and c-Jun-N-terminal kinase (JNK) [37,38]. These signaling cascades are activated in response to extracellular mitogenic and stress stimuli and act to further regulate Smad signaling as well as differentiation, proliferation, cell survival, and apoptosis. Activation of ERK has been shown to increase [39] or decrease [40] Smad signaling depending on the cell type. Conversely, activation of p38 MAPK and JNK typically potentiates TGF-β/Smad effects [41,42]. TGF-β also induces G0/G1 cell cycle arrest independent of Smad proteins via p38 MAPK [43] and has been shown to activate PI3 kinase/Akt, c-Abl, and Rho GTPase signaling cascades [37].

Although the cell signaling underlying AT_2_ activation is less well defined, there is evidence that AT_2_ activation results in attenuation of both canonical (Smad-dependent) and noncanonical (MAPK) TGF-β1 signaling cascades, particularly ERK signaling [44].

### Angiotensin II and AT_1_ expression in fibrosis outside the heart and kidney

Both AT_1_ and AT_2_ have been shown to be upregulated after injury in the heart [45], blood vessels [46], brain [47], nerves [48], and skin [5]. This occurs as early as 24 h after injury and persists for up to 3 months [23].

Enhanced signaling of AngII via AT_1_ in injured tissue has been established in cardiovascular and renal disease as well as Alzheimer’s, Parkinson’s, and stroke [20,45,49]. Additionally, AngII blockade by angiotensin-converting enzyme inhibitors (ACEIs) or AngII receptor blockers (ARBs) has been shown to significantly improve or reverse fibrosis in the skeletal muscle, heart, kidney, liver, and lung [50-55].

Angiotensin II-mediated AT_1_ activation has been shown to play an important role in the development of pulmonary fibrosis. Abdul-Hafez et al. demonstrated that the transition of normal lung fibroblasts to myofibroblasts by TGF-β1 *in vitro* is accompanied by robust expression of angiotensin mRNA, protein, and AngII peptide [56]. In addition, the increased contractility of lung fibroblasts isolated from fibrotic lungs has been shown to be dependent on angiotensin signaling [57].

Paizis et al. demonstrated that following liver injury, there is a marked increase in AT_1_ localized to areas of active fibrogenesis [58]. Expression of AT_1_ has been documented on activated, but not quiescent, hepatic stellate cells which are the key mediator of hepatic fibrosis, suggesting an important role of RAS in chronic liver disease [4]. Suekane et al. conducted a phenotypic analysis of colonic strictures isolated from Crohn’s patients and found that the fibromuscular cells accumulated within strictures strongly expressed AT_1_ [59].

In 2005, Steckelings et al. characterized the distribution of angiotensin receptor within normal and wounded human skin [23]. In normal, unwounded skin, AT_1_ and AT_2_ receptors are expressed on keratinocytes throughout the epidermis but not on dermal fibroblasts, despite the fact that both cell types have comparable levels of mRNA for both receptor subtypes. After wounding, AT_1_ and AT_2_ are upregulated in both epidermis and dermis within 48 h and persist within the scar for up to 3 months.

Hypertrophic scars, keloids, and scleroderma are pathologic fibrotic cutaneous conditions. Although histologically distinct, they all demonstrate increased expression of TGF-β1 and AngII activity [60-62]. Patients with diffuse cutaneous systemic sclerosis (SSc) have significantly elevated serum levels of AngII and cutaneous angiotensinogen expression, which is not expressed in healthy skin [62]. In fact, subcutaneous infusion of AngII has been shown to induce dermal fibrosis in mice, opening up potential for a novel animal model of skin fibrosis [63].

### Antifibrotic effects of AT_1_ inhibition

As previously stated, AngII exerts profibrotic effects via AT_1_ receptor signaling. Over nearly three decades, research has established the therapeutic efficacy of RAS blockade for management of hypertension, heart failure, and renal disease. The body of research focusing on targeting AngII in cardiac and renal fibrosis is extensive, and several thorough reviews have been written on this topic [64-66].

Experimental and clinical studies have provided evidence that targeting angiotensin may be of therapeutic benefit in the management of pulmonary fibrosis. In animal models, inhibition of AT_1_ signaling has been shown to attenuate experimental pulmonary fibrosis induced by bleomycin [67], radiation [68], and hyperoxia [69]. In a pilot clinical study, Couluris et al. evaluated the effect losartan, an AT_1_ antagonist, on idiopathic pulmonary fibrosis progression over 12 months [34]. Preliminary data demonstrated stable or improved pulmonary function testing in 12 of the 17 patients treated with losartan. The authors concluded that losartan is a promising low-toxicity agent for treatment of idiopathic pulmonary fibrosis that requires more extensive evaluation in a placebo-controlled multicenter trial.

The literature also supports a beneficial effect of ACEI/ARBs in preventing or attenuating peritoneal fibrosis that occurs in long-term peritoneal dialysis patients [70]. Recently, losartan was shown to improve fibrosis in a rat model of Crohn’s disease [71]. Despite the large number of animal studies, there is a relative paucity of clinical data. There have been a number of small prospective human studies that have demonstrated a benefit of ACEI/ARB therapy in the treatment of liver fibrosis associated with hepatitis C [69-73]. While a number of clinical studies have demonstrated decreased liver fibrosis with losartan therapy [74,75], the multicenter HALT-C trial showed no significant reduction in the liver fibrosis score in patients on ACEI/ARB therapy [76]. This finding runs counter to the majority of available literature. As suggested by the authors of a recent review on the subject [77], the finding of the HALT-C analysis may be attributable to an increased proportion of diabetics in the ACEI/ARB treatment, a population known to experience more rapid progression of hepatitis C infection.

The potential role of ARB and ACEI in managing cutaneous fibrosis has not been extensively investigated. Marut et al. reported decreased Smad2/3, collagen concentration, and α-SMA expression in a mouse model of systemic sclerosis among animals treated with the ARB irbesartan [78]. In the clinical literature, a handful of case reports describe a subjective incidental improvement in the appearance of keloids or hypertrophic scars following initiation of ACEI or ARB therapy for hypertension [79,80]. Uzun et al. recently compared the effect the oral ACEI enalapril with intralesional triamcinolone in a rabbit ear hypertrophic scar model [81]. They found a modest reduction in scar elevation index in the enalapril group, with a greater reduction in the steroid group.

A number of animal studies have examined the effect of AngII blockade on skeletal muscle fibrosis. Losartan has been shown to reduce fibrosis and restore skeletal muscle strength in animal models of congenital muscular dystrophy and Marfan syndrome, primarily via inhibition of TGF-β1 signaling [55,82]. AT_1_ blockade was also shown to improve muscle regeneration after injury in a mouse gastrocnemius laceration model [83]. Although encouraging, many of these studies employed dosages that were well in excess of the human equivalent dose prescribed for hypertension management [55,83]. To this end, the clinical practicality of AT_1_ blockade for treatment of muscle injury remains unclear.

### Antifibrotic effects of AT_2_ and ACE2/Ang-(1-7)/Mas axis stimulation

Currently, much of the research attention regarding angiotensin has been devoted to exploring function of AT_2_ and the Mas receptor, which, along with ACE2 and angiotensin 1 to 7, comprise the protective arm of the RAS. The recent availability of specific AT_2_ agonists has provided greater momentum in this area, with a number of drugs currently in preclinical or clinical development [18,24].

Stimulation of the ACE2/Ang-(1-7)/Mas axis has been shown to protect against bleomycin-induced pulmonary fibrosis, aspiration-induced lung injury, and severe acute respiratory syndrome (SARS) [84-86]. Wagenaar et al. demonstrated that agonists of Mas and AT_2_ protect against cardiopulmonary disease in rats with neonatal hyperoxia-induced lung injury [87]. Fibroblasts isolated from human fibrotic lung produce significantly more collagen *in vitro* when compared to normal fibroblasts. Uhal et al. demonstrated that this phenomenon is abolished by administration of an ARB [88].

Ang-(1-7) has also been shown to play a protective role in hepatic fibrosis [89]. Infusion of Ang-(1-7) has been shown to reduce fibrosis and proliferation by inhibiting activation of hepatic stellate cells, which are a major fibrogenic cell type in the liver [90].

In skeletal muscle, Ang-(1-7) was shown to decrease fibrosis and increase muscle strength in an animal model of muscular dystrophy [91]. Morales et al. recently demonstrated that this effect was related to attenuation of AngII-induced TGF-β1 expression by Ang-(1-7)/Mas activation [92].

AT_2_ signaling has garnered considerable attention for its potential role in improving peripheral nerve regeneration. Both AT_1_ and AT_2_ receptors are expressed on Schwann cells [93], with a dramatic upregulation of AT_2_ expression observed following injury [94]. Subsequent animal experiments demonstrated that AT_2_ stimulation accelerates axonal regeneration and myelination [94,95]. In addition, the axonal recovery resulted in gain of function in a sciatic nerve injury model in the rat [94]. Most recently, AT_2_ stimulation has been shown to have a beneficial effect in a mouse spinal cord injury model [96].

The beneficial effects of AT_2_ stimulation have also been demonstrated in models of stroke and Alzheimer’s [97], McCarthy et al. demonstrated that AT_2_ stimulation in a rat prior to stroke reduced the severity of neuronal injury in a dose-dependent manner [98].

## Conclusions

The presence of an autologous renin-angiotensin system extends beyond the classically recognized renal and cardiovascular systems and has been demonstrated in almost all tissues of the body. It is now recognized that AngII acts both independently and in synergy with TGF-β to induce fibrosis via the AT_1_ in a multitude of conditions including tubulointerstitial nephritis, myocardial infarction, and systemic sclerosis. Interestingly, recent research has described a paradoxically regenerative effect of the angiotensin system via stimulation of AT_2_. Activation of AT_2_ has been shown to ameliorate fibrosis in animal models of cardiovascular, renal, and neurologic diseases. Manipulation of AngII signaling via AT_1_ blockade and AT_2_ stimulation represent promising therapeutic approaches for inhibiting tissue fibrosis.

## Abbreviations

AngII: Angiotensin II
RAS: Renin-angiotensin system
ECM: Extracellular matrix
α-SMA: Alpha-smooth muscle actin
TGF-β: Transforming growth factor-beta
ACE: Angiotensin-converting enzyme
AT_1_: Angiotensin II type 1 receptor
AT_2_: Angiotensin II type 2 receptor
Ang1-7: Angiotensin (1-7)
NF-κB: Nuclear factor kappa-light-chain-enhancer of activated B cells
MMP: Matrix metalloproteinase
TIMPs: Tissue inhibitor metalloproteinase
CTGF: Connective tissue growth factor
LAP: Latency-associated peptide
TβRII: TGF-β1 type II receptor
TβRI: TGF-β1 type I receptor
ERK: Extracellular signal-related kinase
JNK: c-Jun-N-terminal kinase
ARBs: AngII receptor blockers
ACEi: Angiotensin-converting enzyme inhibitor
